# PD-1/PD-L1 blockade in cancer treatment: perspectives and issues

**DOI:** 10.1007/s10147-016-0959-z

**Published:** 2016-02-22

**Authors:** Junzo Hamanishi, Masaki Mandai, Noriomi Matsumura, Kaoru Abiko, Tsukasa Baba, Ikuo Konishi

**Affiliations:** Department of Gynecology and Obstetrics, Kyoto University Graduate School of Medicine, 54 Kawahara-cho, Shogoin, Sakyo-ku, Kyoto, Kyoto 606-8507 Japan; Department of Obstetrics and Gynecology, Kinki University Faculty of Medicine, Osaka, Japan

**Keywords:** PD-1, PD-L1, Nivolumab, Immunotherapy, Biomarker, Value

## Abstract

Recent studies showed that tumor cells ‘edit’ host immunity in several ways to evade immune defenses in the tumor microenvironment. This phenomenon is called “cancer immune escape.” One of the most important components in this system is an immunosuppressive co-signal (immune checkpoint) mediated by the PD-1 receptor and its ligand, PD-L1. PD-1 is mainly expressed on activated T cells, whereas PD-L1 is expressed on several types of tumor cells. Preclinical studies have shown that inhibition of the interaction between PD-1 and PD-L1 enhances the T-cell response and mediates antitumor activity. Several clinical trials of PD-1/PD-L1 signal-blockade agents have exhibited dramatic antitumor efficacy in patients with certain types of solid or hematological malignancies. In this review, we highlight recent clinical trials using anti-PD-1 or anti-PD-L1 antibodies against several types of malignancies, including a trial conducted in our department, and describe the clinical perspectives and issues regarding the PD-1/PD-L1 blockade in cancer treatment.

## Introduction

Tumor cells have acquired several ways to escape from host immunity in the tumor microenvironment, called cancer immune escape via cancer immunoediting process [1]. During the past two decades, several studies of cancer immune escape revealed that one of the most important components of the underlying mechanism is an immunosuppressive co-signal (immune checkpoint) mediated by programmed cell death-1 (PD-1)/PD-1 ligand 1 (PD-L1) in the tumor microenvironment.

**Fig. 1:**
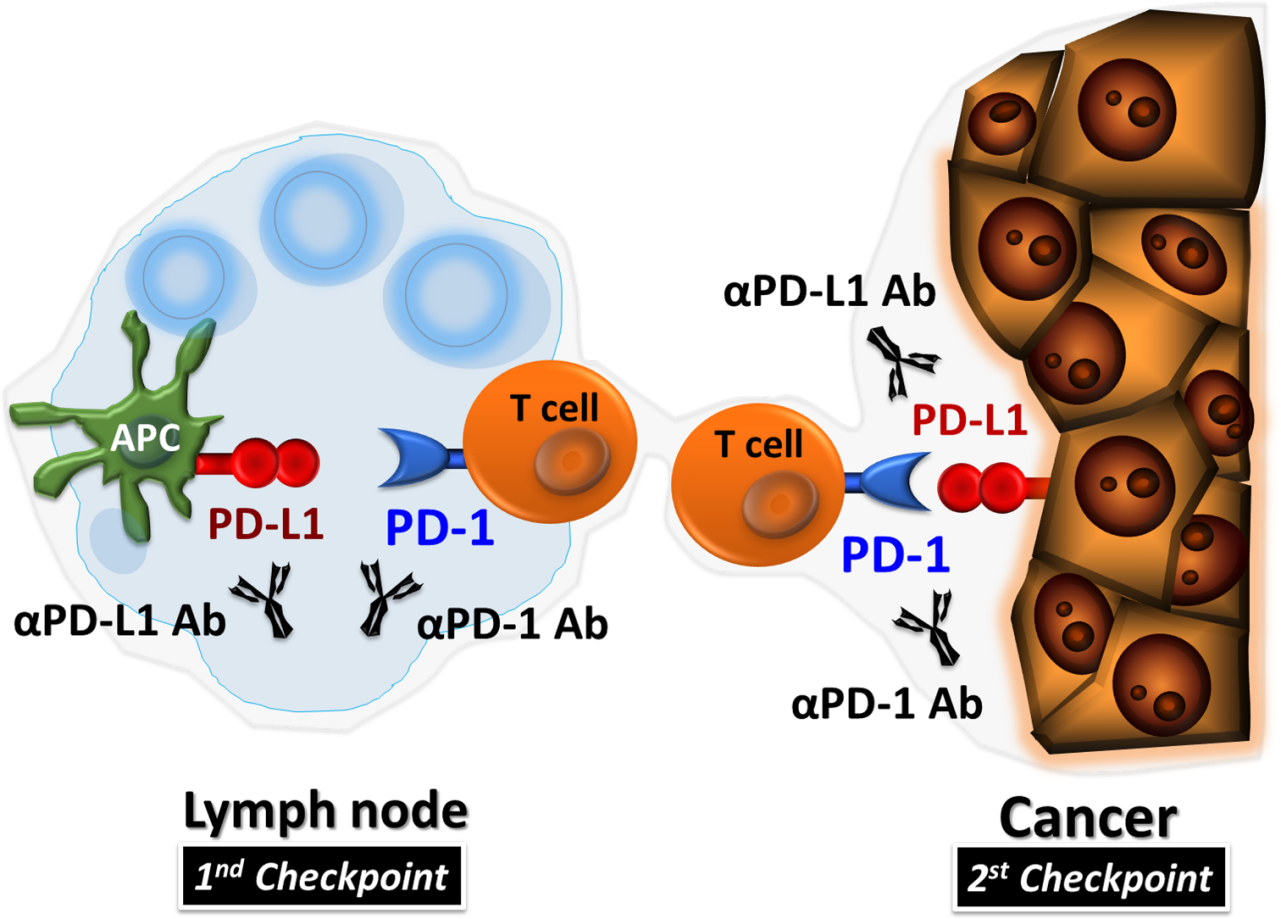
Programmed death (PD)-1 inhibitors in cancer. PD-1 inhibitors (anti-PD-1 antibody and anti-PD-L1 antibody) block PD-1/PD-L1 signaling and induce anti-tumor immune reactivation at two checkpoints: cognitive phase (lymph node) and effector phase (tumor microenvironment). *αPD-1 Ab* anti–PD-1 antibody, *αPD-L1 Ab* anti–PD-L1 antibody

PD-1 was discovered by Tasuku Honjo and colleagues at Kyoto University in 1992 [2]. PD-1 expressed on T cells negatively regulates their antitumor effect [3–5]. PD-L1 engages with PD-1 to inhibit proliferation and cytokine production by T cells [6, 7]. Several studies showed that under normal physiological conditions, PD-L1 is expressed in human tonsil, placental syncytiotrophoblast, monocyte, and lung, where it is involved in immune tolerance [8, 9]. Several preclinical reports also showed that inhibition of the interaction between PD-1 and PD-L1 enhances the T-cell response and mediates antitumor activity [3–5, 10]. Additionally, PD-L1 is expressed on various human cancers, including urothelial cancers, gastrointestinal cancers, lung cancer, breast cancer, melanoma, and ovarian cancer, as well as on tumor-infiltrating immune cells in the tumor microenvironment [8, 9, 11–18]. Therefore, to provide proof of principle that the PD-1/PD-L1 blockade is effective against cancer, efforts were made to develop PD-1 inhibitors (anti-PD-1 or anti-PD-L1 antibody) for the treatment of human cancers (Fig. 1). The clinical trials performed to date have been highly successful.

In this review, we summarize recent clinical applications of PD-1/PD-L1 blockade in cancer treatment, as well as discuss some pertinent perspectives and issues leading to further effective clinical application of PD-1 inhibitors to various malignant tumors in the near future.

## Clinical applications of PD-1 inhibitors in cancer

In light of fundamental research, clinical studies using PD-1 pathway inhibitors against treatment-resistant solid tumors were initiated in the United States in 2006 [19]. To date, at least 200 such clinical studies have been carried out using nine types of antibody in at least 20 types of cancer, including both solid and hematological tumors; the total number of subjects worldwide is more than 20,000 (Table 1).

**Table 1:** Programmed death (PD)-1 inhibitors (anti-PD-1 antibodies and anti-PD-L1 antibodies) in clinical testing

Target	Agent	IgG class	Company	Approved
PD-1	Nivolumab (Opdivo^®^, BMS-936558, MDX1106)	Human IgG4	Bristol-Meyers Squibb/Ono	Melanoma^1^ Lung cancer^2^
	Pembrolizumab (Keytruda^®^ MK-3475, lambrolizumab)	Humanized IgG4	Merck	Melanoma^3^ Lung cancer^4^
	Pidilizumab (CT-011)	Humanized IgG1k	Cure Tech	
	AMP-224	PD-L2 IgG2a fusion protein	Amplimmune/GlaxoSmith Klein	
	AMP-514 (MEDI0680)	PD-L2 fusion protein	Amplimmune/GlaxoSmith Klein	
PD-L1	BMS-936559 (MDX1105)	Human IgG4	Bristol-Meyers Squibb	
	Atezolizumab (MPDL3280A)	Human IgG1k	Roche/Genentech	
	Durvalumab (MEDI4736)	Human IgG1k	MedImmune/AstraZeneca	
	Avelumab (MSB0010718C)	Human IgG1	Merck Serono/Pfizer	

^1^ Melanoma: approved in USA, EU, and Japan

^2^ Lung cancer: squamous cell lung cancer in USA an EU; non small lung cancer in Japan

^3^ Melanoma: approved in USA, EU

^4^ Lung cancer: PD-L1+ non small lung cancer in USA

In 2010, the first phase I clinical trial of an anti-PD-1 antibody, nivolumab, was conducted in 39 patients with treatment-refractory solid tumors such as advanced melanoma, non-small cell lung cancer (NSCLC), renal cell carcinoma (RCC), prostate cancer, and colorectal cancer (CRC). The response rate (RR) was 7.7 %, including one durable complete response in CRC and two partial responses in melanoma and RCC. Only one serious adverse event (inflammatory colitis) was observed, so nivolumab was considered to be well tolerated [19].

Subsequently, in 2012, a phase I study of nivolumab was carried out in a total of 296 patients with NSCLC, melanoma, or RCC; the mean RR values were 18 %, 28 %, and 27 %, respectively. The most frequent adverse effects (AEs) were rash, diarrhea, and itching (in 12 %, 11 %, and 9 % of subjects, respectively); AEs that occurred in ≥1 % of subjects at grade 3 or 4 were diarrhea, hepatic dysfunction, and pneumonia. In particular, three subjects died of pneumonia; consequently, a cautionary note about immunological side reactions has been reported [20]. In addition, in a follow-up study, durable antitumor responses were observed, with overall survival (OS) of 9.9, 22.4, and 16.8 months for patients with NSCLC, RCC, and melanoma, respectively [21]. These studies constituted a turning point in the expansion of clinical applications of PD-1 inhibitors, and their results were reported in important publications that made major contributions to the ongoing rapid progress with these agents. Representative clinical trials in different types of cancer are summarized here.

### Melanoma

Representative phase III clinical trials with anti-PD-1 antibodies, nivolumab and pembrolizumab, in patients with melanoma have led to the separate approval of both drugs. The first phase III trial with nivolumab as a first-line therapy was conducted in 418 treatment-free patients with metastatic melanoma lacking a mutation in BRAF. Patients were randomized to receive either nivolumab or chemotherapy with dacarbazine. The overall survival rate at 1 year was 73 % for the nivolumab patients versus 42 % in dacarbazine. Similarly, median progression-free survival was 5.1 months in the nivolumab group versus 2.2 months in the dacarbazine group. The objective response rate was 40 % in the nivolumab group as compared with 14 % in the dacarbazine group [22]. The second randomized phase III trial compared nivolumab to chemotherapy (dacarbazine or carboplatin/paclitaxel) in 405 patients with advanced metastatic melanoma [23]. In contrast to the previous trial, these patients had all been previously treated with ipilimumab, and a minority had also received a BRAF inhibitor. ORR was threefold higher in the nivolumab group than in the chemotherapy group (32 % vs. 11 %).

In another randomized phase III trial, 834 patients were with advanced melanoma were divided equally among three groups: pembrolizumab every 2 weeks, pembrolizumab every 3 weeks, or ipilimumab every 3 weeks. The estimated 6-month progression-free survival rates were 47 %, 46.4 %, and 26.5 %, respectively, and the estimated overall 1-year survival rates were 74 %, 68 %, and 58 %, respectively. The response rate was improved with pembrolizumab administered every 2 weeks (33.7 %) and every 3 weeks (32.9 %), as compared with ipilimumab (11.9 %) [24].

In 2014, the U.S. Food and Drug Administration (FDA) approved nivolumab and pembrolizumab for the treatment of advanced melanoma (both are limited to be approved for melanoma refractory to ipilimumab and with BRAF inhibitors, if the tumor harbors a BRAF mutation). Later, in 2015, the FDA additionally expanded an approval of nivolumab with BRAF wild-type and pembrolizumab for first treatment of unresectable or metastatic melanoma, each.

Similar to anti-PD-1 antibodies, anti-PD-L1 antibodies in phase I clinical trials for melanoma have shown promising antitumor activity. The first phase I trial of an anti-PD-L1 antibody, BMS-936559, yielded a RR of 17 % in 52 patients with advanced melanoma [25]. Other anti-PD-L1 antibodies, atezolizumab and durvalumab, in ongoing phase I trials have also exhibited clinical activities in patients with NSCLC. The RR values of these antibodies and the sample sizes of the respective studies are as follows: atezolizumab, 30 % (*n* = 43) [26]; durvalumab, 13 % (*n* = 8) [27].

### Lung cancer

Representative phase II or III clinical trials with anti-PD-1 antibodies, nivolumab and pembrolizumab, in patients with non-small cell lung cancer (NSCLC) are described here. A phase II single-arm trial of nivolumab in 117 patients with advanced refractory squamous NSCLC yielded an RR of 14.5 %; median duration of response had not been reached at the time of analysis. Although the RR was relatively low, there is currently no standard of care in this third-line setting. Nivolumab has also exhibited promising effects as a second-line treatment for squamous NSCLC [28].

In a randomized phase III trial comparing nivolumab (*n* = 131) to docetaxel (*n* = 129) in patients with squamous NSCLC who had disease recurrence after one prior platinum-containing regimen, the median OS was 9.2 months in the nivolumab group versus 6.0 months in the docetaxel group. At 1 year, the overall survival rate was 42 % with nivolumab versus 24 % with docetaxel. The risk of death was 41 % lower with nivolumab than with docetaxel (HR, 0.59; *P* < 0.001); the response rate was 20 % with nivolumab versus 9 % with docetaxel (*P* = 0.008), and the median progression-free survival (PFS) was 3.5 months with nivolumab versus 2.8 months with docetaxel (HR, 0.62; *P* < 0.001) [29]. Based on these interim data, in 2014 the FDA expanded the approval of nivolumab to include squamous NSCLC with disease recurrence after one prior platinum-containing regimen.

In a randomized phase III trial comparing nivolumab (*n* = 292) to docetaxel (*n* = 290) in non-squamous NSCLC after failure of platinum-doublet chemotherapy, OS was 12.2 months in the nivolumab group versus 9.4 months in the docetaxel group. At 1 year, the overall survival rate was 51 % with nivolumab versus 39 % with docetaxel [30]. Based on these data, in late 2015 the FDA significantly expanded the approval of nivolumab to previously treated metastatic NSCLC. Additionally, in the first-line setting, a randomized phase III trial is currently underway to compare nivolumab to investigator’s choice chemotherapy in patients with previously treated or untreated NSCLC whose tumors express PD-L1 [31].

Four anti-PD-L1 antibodies in the early phase of clinical trials have also demonstrated clinical activity in patients with NSCLC. The RR values of these antibodies and the sample sizes of the respective studies are as follows: BMS-936559, 10 % (*n* = 49) [25]; durvalumab, 16 % (*n* = 58) [32]; atezolizumab, 21 % (*n* = 53) [27]; avelumab, 12 % (*n* = 184) [33].

### Renal cell carcinoma

In a phase II clinical trial of nivolumab in 168 patients with metastatic renal cell carcinoma (RCC) patients who had previously received vascular endothelial growth factor (VEGF) pathway inhibitors, RR was 21 % [34]. In a randomized phase III trial of nivolumab in 821 patients with advanced clear cell RCC, the median OS was 25.0 months with nivolumab and 19.6 months with everolimus. RR was higher with nivolumab than with everolimus (25% vs. 5 %), whereas the median PFS was 4.6 months with nivolumab and 4.4 months with everolimus [35]. Based on these data, in late 2015 the FDA approved nivolumab for RCC patients who had received previous treatment with antiangiogenic therapy in late 2015.

Anti-PD-L1 antibodies in phase I clinical trials for RCC have also demonstrated clinical activity in patients with RCC. The RR values of these antibodies and the sample sizes of the respective studies are as follows: BMS-936559, 12 % (*n* = 17) [25] and atezolizumab, 14 % (*n* = 56) [27] in patients with RCC.

### Ovarian cancer

Based on our clinical studies of cancer immune escape in ovarian cancer [14, 36, 37], we conducted the first principal investigator-initiated two-cohort (1 or 3 mg/kg, *n* = 10 each), phase II clinical trial of nivolumab in 20 patients with platinum-resistant recurrent ovarian cancer [38]. The trial was conducted in collaboration with Professor Honjo, who discovered the PD-1 gene [1]. RR at 3 mg/kg was 20 %, including 2 cases of a durable complete response (CR). Among all 20 patients, RR was 15 % and DCR was 45 %; median PFS and OS were 3.5 and 20.0 months, respectively [39]. In our ongoing follow-up study subsequent to this trial, a durable antitumor response with nivolumab has been observed in 2 patients with a CR for more than 1 year [40]. After completing the 1-year nivolumab treatment according to our study design, these 2 patients have each survived without any disease progression for more than 1 year, with no adjuvant antitumor treatment [41].

Later, the interim results became available from two other phase Ib clinical trials with anti-PD-1 antibody [25] and anti-PD-L1 antibody (avelumab). In the trial of pembrolizumab in 26 patients with PD-L1+ advanced ovarian cancer, RR was 11.5 % [42]. In the trial of avelumab in 75 patients with recurrent or refractory ovarian cancer, RR was 10.7 % [43].

### Head and neck cancer

The anti-PD-1 antibody pembrolizumab exhibited antitumor activity in patients with refractory squamous cell carcinoma of the head and neck cancer (SCCHN), whose tumors expressed ≥1 % PD-L1 (RR = 20 % in 56 patients) [44]. Anti-PD-L1 antibodies in phase I clinical trials for SCCHN have also exhibited clinical activity. The RR values of these antibodies and the sample sizes of the respective studies are as follows: durvalumab, 14 % (*n* = 22) [45]; atezolizumab, 17 % (*n* = 6) [27].

### Bladder cancer

In a phase I clinical trial of pembrolizumab in 29 patients with advanced bladder cancer patients whose tumors expressed ≥1 % PD-L1, RR was 24 %, including three cases of CR [46]. Another anti-PD-L1 antibody, atezolizumab, yielded a RR of 26 % in 68 patients with advanced bladder cancer [47]. Based on these findings, in 2014 the FDA set the ‘Breakthrough’ designation for the clinical development of atezolizumab for treatment of advanced bladder cancer.

### Gastric cancer

In a phase I trial of pembrolizumab in 39 patients with advanced gastric cancer, RR was 22 %. Median time to response was 8 weeks, with a median response duration of 24 weeks. The 6-month PFS and OS rates were 24 % and 69 %, respectively [48].

### Colon cancer

In a phase II clinical trial of pembrolizumab in 50 patients with colon cancer, RR was higher in the mismatch repair-deficient group (*n* = 25) than in the mismatch repair-proficient group (*n* = 25), 62 % versus 0 % [49] (see “Clinical perspectives and issues of PD-1 inhibitors” for details).

### Esophageal cancer

In a phase I study of pembrolizumab in 22 patients with esophageal cancer, RR was 23 %, including 3 (11 %) cases of CR [50].

### Hepatocellular cancer

In a phase I/II trial of nivolumab in 42 patients with hepatocellular carcinoma, CR was reported in 2 patients (5 %) and PR in 7 patients (18 %) for an overall objective RR of 23 %. Overall survival (OS) rate at 6 months was 72 % [51].

### Breast cancer

In a phase 1b trial of pembrolizumab in 25 patients with advanced triple-negative breast cancer (TNBC) whose tumors expressed ≥1 % of PD-L1, RR was 12 % [52]. A phase Ia study of the anti-PD-L1 antibody atezolizumab in 27 patients with metastatic TNBC yielded a RR of 19 %, including 1 CR and 4 PRs [53].

### Hematological malignancies

Nivolumab has shown dramatic antitumor effects in patients with relapsed or refractory Hodgkin’s lymphoma (HL) (*n* = 23), with a RR of 87 % and a DCR of 100 %, including CR in 20 % of patients [54].

Pembrolizumab also yielded a RR of 53 % in 15 patients with pre-treated HL, including CR in 20 % of patients [55]. In a phase I trial of nivolumab in patients with non-HL, diffuse follicular lymphoma (*n* = 10) or large B-cell lymphoma (*n* = 11), RR values were 40 % and 36 %, respectively [56].

## Clinical perspectives and issues of PD-1 inhibitors

The accumulated data from clinical trials for solid tumors revealed that the antitumor response rate of PD-1 inhibitors seems not so high. Therefore, there is an urgent need to resolve several issues related to PD-1 inhibitors (Fig. 3). First, to enhance the antitumor effect of PD-1 inhibitors, we have to find the best combination therapy with other antitumor therapy such as chemotherapy, targeted therapy, radiotherapy, or other immunotherapy. Second, because PD-1 inhibitors are very expensive, it is necessary to identify predictive biomarkers that allow selection of appropriate patients. Third, we must learn how to manage severe immunological side effects. Last, we have to investigate the most valuable application of PD-1 inhibitors.

### Development of effective combination therapies

Clinical trials of combination treatments incorporating PD-1 inhibitors with conventional chemotherapies [57], molecular targeted drugs such as PARP inhibitors (olaparib and cediranib) for solid tumors [58], or multi-kinase inhibitor (sunitinib) for RCC [59], focal radiation therapy, and cancer immunomodulators are now underway. In particular, therapy using double-checkpoint inhibitors in combination with nivolumab and ipilimumab for the treatment of advanced melanoma led to longer PFS than either agent alone [60]. However, the frequency of grade 3 or 4 immune-related AEs was also amplified to more than 50 %. Similar combinations are now being clinically applied to diverse cancer types, including RCC [61], NSCLC [62], or ovarian cancer [63].

Next-generation clinical trials are also underway for PD-1 inhibitors in combination with other immune modulators such as anti-lymphocyte activation gene 3 (LAG3) antibody for solid tumors [64], anti-killer inhibitory receptor (KIR) antibody, lirilumab, for solid tumors [65], anti-OX40 agonistic antibody, MEDI6383, for solid tumors [66], anti-4-1BB agonistic antibody, urelumab, for solid tumors and B-cell non-Hodgkin lymphoma [67], and GM-CSF-producing and CD40L-expressing bystander cell line (GM.CD40L) vaccine for NSCLC [68].

### Identification of biomarkers

The identification of predictive biomarkers of PD-1 inhibitors is a crucial next step for advancing the applications of these drugs. Potential predictive biomarkers of antitumor response to PD-1 inhibitors can be considered in either tumor cell-related factors or host immunological factors. Recent studies identified several candidate biomarkers: expression of PD-L1 in tumor cells and tumor-infiltrating lymphocytes, frequencies of mutations in tumor cells, and diversity of tumor antigen-specific T cells (T-cell repertoire). All these candidates are regarded as viable prospects, although some concerns are associated with each of them.

#### PD-L1 and TILs

Since the first clinical trial of nivolumab in 2010 [19], several reports of clinical trials of PD-1 inhibitors have shown that the therapeutic efficacy of PD-1 inhibitors is modestly correlated with PD-L1 expression in tumors [20, 22, 27, 47, 48, 69, 70]. In addition, with respect to tumors such as melanoma and bladder cancer, not only tumor cells, but also the number of infiltrating T cells and the proportion of T cells positive for PD-L1 or PD-1 expression, can be used as indices of therapeutic efficacy [27, 47, 71]. In other words, the effects of PD-1 pathway-inhibiting drugs occur when tumor cells and immune cells are present at a particular locus and the cancer-immunosuppressive state mediated by the PD-1 pathway has been established. In future, in addition to follow-up studies, diagnostic-grade evaluation and accuracy control of PD-L1 expression will also be required. Therefore, there is a need to establish clearer and more accurate analysis methods [72].

On the other hand, it is probable that PD-L1 expression by tumors will change during treatment in response to the immune state and the administration of therapeutic drugs [27, 73–75]. Therefore, it is necessary to investigate the influence of parameters such as the timing and locus of sample collection by biopsies and surgery; whether samples are embedded in paraffin or frozen; and evaluation methods such as immunostaining, quantitative polymerase chain reaction (PCR), and Western blotting. In addition, in cases of tumors within an abdominal cavity, whether primary, secondary, or metastatic, it is often difficult to carry out biopsies from the body surface. Consequently, various issues must be investigated and analyzed, such as the different sampling modes needed to collect different types of tumor tissue.

Furthermore, since in the case of nivolumab for Hodgkin’s lymphoma patients, the response rate was 87 % and the disease-control rate was 100 % [54], biomarkers per se are not useful in such a type of tumor for which efficacy has been demonstrated. On the other hand, in a phase I/II nivolumab study in SCLC patients [76], a phase III nivolumab study in lung squamous cell carcinoma patients [29], and a phase III study of nivolumab plus ipilimumab combination therapy in melanoma patients [60], no significant correlations were observed between PD-L1 expression and therapeutic efficacy. However, in the nivolumab plus ipilimumab combination therapy study, a subanalysis did reveal correlations between PD-L1 expression and therapeutic efficacy in the nivolumab monotherapy group. Therefore, it might be useful to establish a paradigm for therapy selection in which nivolumab monotherapy is used when a tumor is positive for PD-L1 expression, but combination therapy is used when it is negative. In all these studies, therapeutic efficacy was observed even with PD-L1-negative patients, and it is therefore probably inappropriate to select patients solely on the basis of whether they express PD-L1 [60]. Thus, there is a demand for immunologically individualized treatment methods that can be selected accurately, with consideration given to other factors such as cancer type and histological subtype.

#### Relationship between PD-1 signal and genomic mutation

**Fig. 2:**
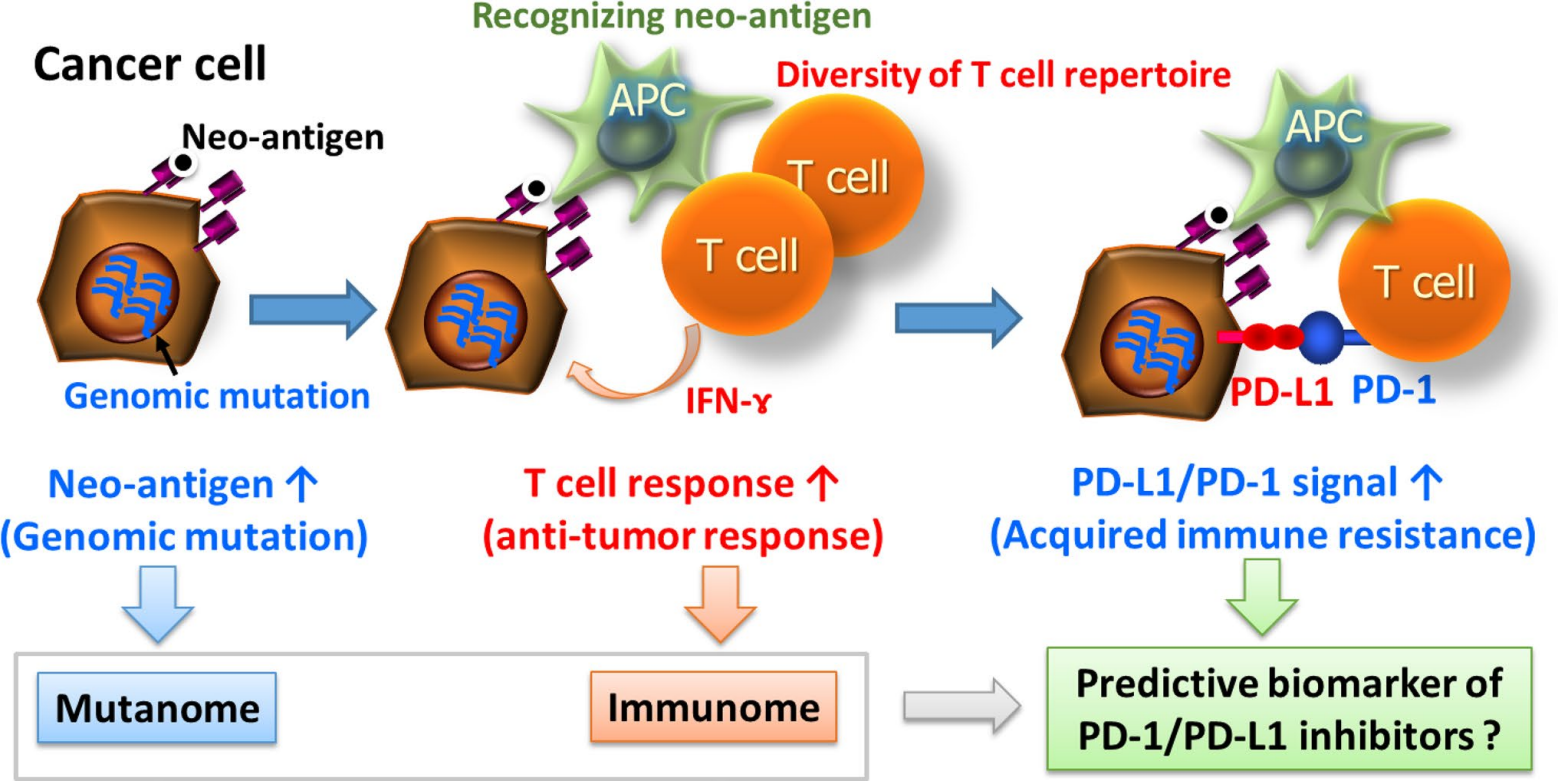
Relationship between PD-1 signal and genomic mutation. Mutated neo-antigens are expressed on the surface of a cancer cell in response to genomic mutation and amplification of a cancer cell. Recognition of a neo-antigen as a foreign body by an antigen-presenting cell (APC) induces a T-cell response, and consequently the activated T cell releases interferon (IFN)-γ. A cancer cell that is exposed to IFN-γ expresses PD-L1, thereby establishing an acquired immune resistance. In this type of tumor microenvironment, a PD-1 pathway inhibitor should be effective; thus, genome-wide mutation analysis (i.e., mutanome analysis) of cancer cells using next-generation sequencing technology and diversity analysis of the T-cell repertoire (i.e., the immunome) have attracted attention as strategies for identification of predictive biomarkers. *APC* antigen-presenting cell

Rapid progress has also been made in reverse-translational research using specimens from patients who received immune checkpoint inhibitors. In addition, recent progress in genomic analysis using next-generation sequencing techniques has enabled comprehensive detection of mutations in cancer tissue. According to *The Cancer Genome Atlas*, which covers 7042 tumor samples from 30 cancer types, the frequency of somatic gene mutations is highest in melanoma, followed by lung squamous cell carcinoma, lung adenocarcinoma, bladder cancer, stomach cancer, and esophageal adenocarcinoma [77]. PD-1-inhibitors are expected to be therapeutically effective in cancer types with high somatic gene mutation frequencies [78, 79]. In other words, cancer types with numerous mutations necessarily also express numerous mutant cancer antigens, and immunosuppression mediated by the PD-1 pathway occurs in the presence of numerous T cells that specifically recognize those mutant antigens. Thus, PD-1 pathway inhibitors represent a promising class of drugs for use in such cases (Fig. 2). This position supports the findings of fundamental research using a mouse model that demonstrated the existence of mutant cancer antigen-specific T cells, as well as reactivation of these cells by anti-PD-1 antibodies and anti-CTLA-4 antibodies (CTLA-4 is a receptor for the same immunosuppressive signals as PD-1) [80].

Snyder et al. performed whole-exome sequencing of tumors from 64 melanoma patients who had been treated with the anti-CTLA-4 antibody ipilimumab or tremelimumab. The results revealed durable clinical efficacy in 11 subjects, and the mutation levels in these patients were significantly elevated [81]. Because neither of these factors is sufficient as a predictive marker for treatment, genome-wide somatic cell neo-epitope analysis and HLA analysis were carried out, resulting in identification of a neo-epitope candidate that is specifically expressed in tumors against which anti-CTLA-4 antibodies are therapeutically effective. This neo-epitope was validated in a dataset comprising 39 melanoma patients. In addition, the neo-epitope activated T cells derived from patients who received ipilimumab, demonstrating the usefulness of mutation analysis by whole-exome sequencing, as well as neo-epitope analysis, in predicting the therapeutic efficacy of anti-CTLA-4 antibodies.

In addition, Rizvi et al. carried out whole-exome sequence analysis of tumors in NSCLC patients treated with the anti-PD-1 antibody pembrolizumab. The results revealed that when numerous non-synonymous mutations were present, there were correlations between response to treatment, durable clinical benefit (i.e., partial response or stable disease for at least 6 months), and recurrence-free survival rate [82]. Similarly, correlations were observed between therapeutic efficacy and a set of genes that is upregulated in smokers, neo-antigen count, and mutations in the DNA-repair pathway, all of which are linked to the mutation level. Furthermore, some studies have described patients who exhibit neo-antigen-specific T-cell immune responses that increase with tumor contraction upon treatment with pembrolizumab. Therefore, it is possible that the efficacy of pembrolizumab treatment against lung cancer is determined by the genomic landscape of the cancer.

In addition, Le et al. found that in a phase II tremelimumab study carried out previously in CRC patients, 1 of 47 subjects exhibited a partial response. In addition, in a phase I study in which the anti-PD-L1 antibody MPDL3280A was administered to 20 subjects, 1 CRC patient with deletion of a mismatch repair (MMR) gene exhibited a partial response [49]. Therefore, the anti-PD-1 antibody pembrolizumab was administered to three cohorts, A, B, and C, respectively, comprising 25 CRC patients with MMR deletion, 25 CRC patients with normal MMR, and 21 patients with cancers other than CRC with MMR deletion. The therapeutic efficacy was very high in the CRC patients with MMR deletion, with a response rate of 62 % and a disease-control rate of 92 %. By contrast, in the 25 CRC patients with normal MMR, the efficacy was very low, with a response rate of 0 % and a disease-control rate of 16 %. Furthermore, in the subjects with non-CRC cancers with MMR deletions, the response rate and disease-control rate were 60 % and 70 %, respectively, suggesting the possibility that MMR deletion is a predictive factor for the therapeutic efficacy of anti-PD-1 antibody, pembrolizumab.

In the manner already described, a search for biomarkers was recently carried out via comprehensive mutation analysis of the cancer genome using next-generation sequencing technology. This approach is termed mutanome analysis when it involves genome-wide mutation analysis of cancer cells, and immunome analysis when it involves a comprehensive exploratory analysis related to tumor immunology; the latter includes diversity analysis of a T-cell repertoire, microarray analysis, and protein analysis. By making extensive use of these techniques, high-throughput extraction of markers can be carried out more effectively, and rapid progress is being made in verification techniques for validation studies even in the field of cancer immunology (Fig. 2).

#### Integrating several biomarkers in an algorithm

**Fig. 3:**
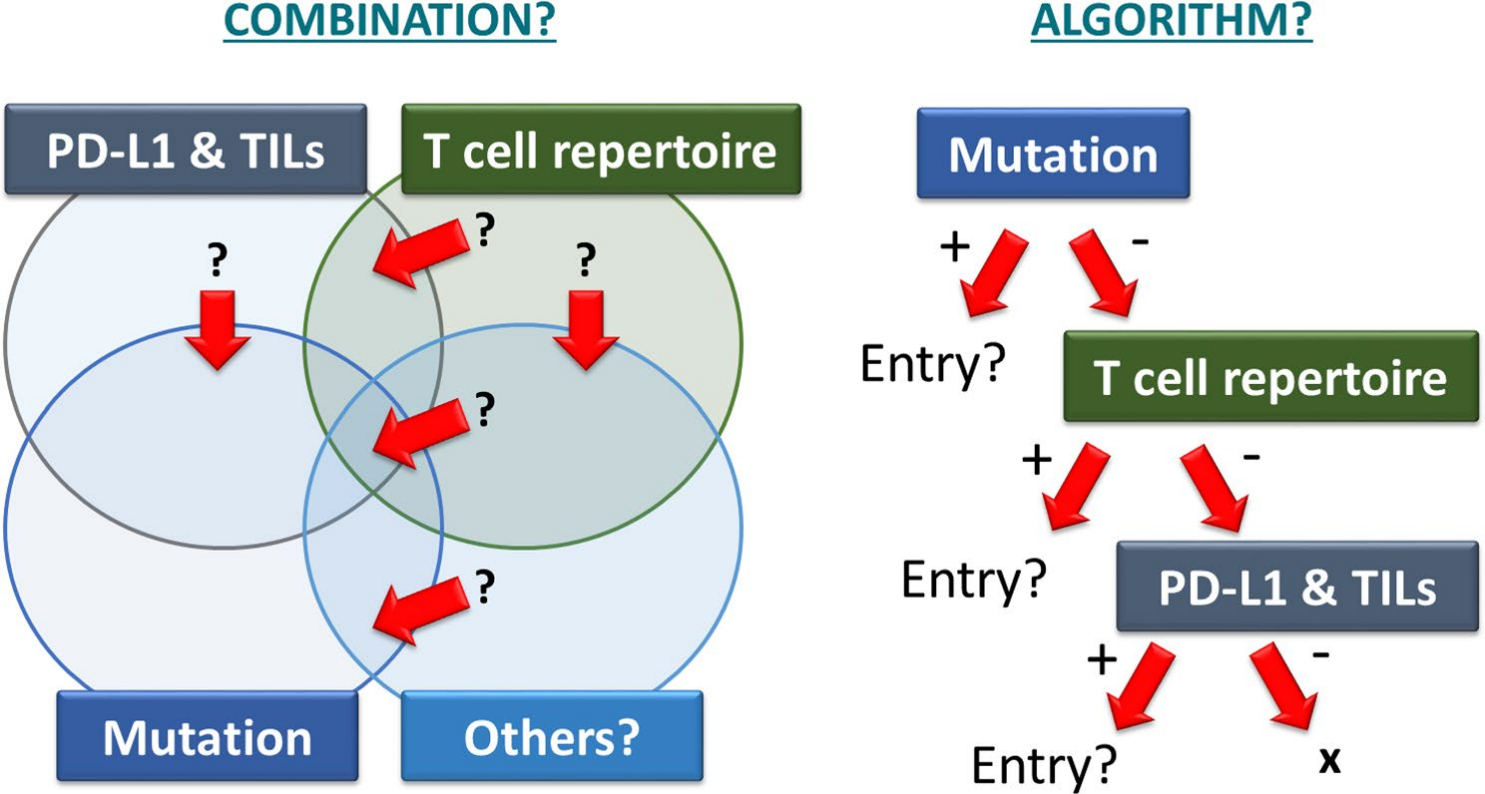
Identification of the best biomarker strategy. A combination or algorithm of biomarkers may be needed. For example, it is important to understand whether it will be necessary to use a combination of biomarkers, e.g., PD-L1+ tumor cells, PD-L1+, and/or PD-1+ tumor-infiltrating lymphocytes, frequencies of neo-antigens and tumor mutations, and diversity of the T-cell repertoire. Additionally, more candidate predictive biomarkers will be identified in the future. Therefore, development of an algorithm or combination of these biomarkers should be a high priority for future work

As already mentioned, several candidates for predictive biomarkers were recently identified. Further study is necessary to improve our understanding of their clinical significance, however, as the best strategy may involve more than one biomarker. Therefore, biomarker combinations and algorithms may be required. For example, it will be important to determine whether it is necessary to use combinations of biomarkers such as PD-L1+ tumor cells, PD-L1+ and PD-1+ tumor-infiltrating lymphocytes, frequencies of neo-antigens and tumor mutations, and the diversity of the T-cell repertoire. Additionally, more candidate predictive biomarkers will be identified in the future. Therefore, the development of useful algorithms or combinations of these biomarkers is a high priority for future work (Fig. 3).

### Immunological side effects

Common drug-related adverse events (AEs) of both anti-PD-1 and anti-PD-L1 antibodies include fatigue, rash, diarrhea, pruritus, decreased appetite, arthralgia, and nausea [20, 22, 23, 29, 83–87]. In particular, immune-related AEs (irAEs) such as dermatitis, colitis, hepatitis, vitiligo, and thyroiditis have been reported, and about 10 % of patients develop grade 3 or 4 irAEs. Rare (<10 %) but life-threatening irAEs include pneumonitis (including acute interstitial pneumonia/acute respiratory distress syndrome), colitis with gastrointestinal perforation, infusion reaction and anaphylactic shock, type 1 diabetes, severe skin reactions, immune thrombocytopenia, neutropenia and sepsis risk after corticosteroid therapy, encephalopathy and neurological sequelae, Guillain–Barré syndrome, myelitis and motor sequelae, myocarditis and cardiac insufficiency, and acute adrenal insufficiency and nephritis [83, 84]. In the first phase II trial of nivolumab, grade 1 or 2 pneumonitis in 6 of 296 patients was reversible upon discontinuation of treatment and administration of glucocorticoid, but 3 of 296 patients (1 %) died of pneumonitis. On the basis of these findings, guidelines and specific algorithms for identification, early intervention, and management of irAEs have been developed [20, 25]. In the clinical setting, if severe irAEs occur, clinicians should perform a timely evaluation to confirm the diagnosis, and if necessary, admit the patient to the hospital and treat with intravenous corticosteroids without hesitation. Moreover, if corticosteroids are not effective, clinicians should consider using additional immunosuppressive drugs such as anti-tumor necrosis factor (TNF) antibody (i.e., infliximab) [83].

Although irAEs can develop at any time, the majority of immune toxicities of nivolumab occur within the first 4 months [84, 85]. The median time to onset of irAEs tends to differ depending on the type of toxicity, and can be roughly classified as early (<2 months) or late (>2 months). Early toxicities include skin (5 weeks), gastrointestinal (7 weeks), and hepatic (7 weeks), whereas late toxicities include pulmonary (9 weeks), endocrine (10 weeks), and renal (15 weeks). However, clinicians should keep in mind that all types of irAEs can occur at any time [84, 87–89].

Finally, we, as gynecologists, believe that we should investigate the gender differences in frequency and severity of side effects and evaluate the risk of infertility for younger men or women who receive PD-1 inhibitors. Currently, very little is known about the influence of PD-1 inhibitors on human fertility or pregnancy (Fig. 3). The drug package insert of nivolumab [86] describes embryofetal toxicity in the cynomolgus monkey and advises females with reproductive potential to use effective contraception during treatment with nivolumab and for at least 5 months after the last dose.

### The value of PD-1 inhibitors

**Fig. 4:**
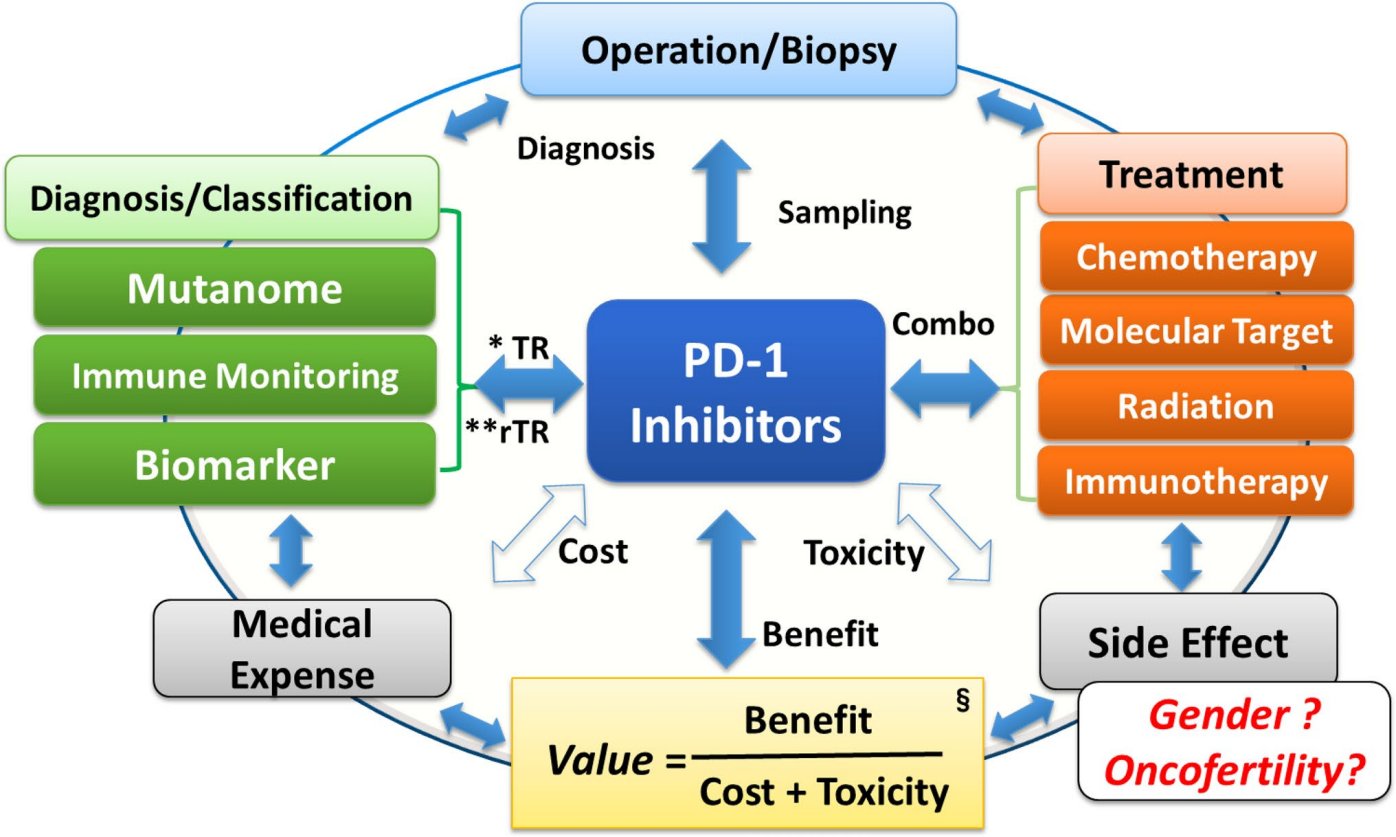
Perspectives and issues regarding PD-1 inhibitors. *Asterisk TR* translational research, *double asterisk* *rTR* reverse translational research. *Section sign* See reference [89]

On the other hand, the American Society of Clinical Oncology increasingly emphasizes the value of anticancer treatments, referring to their benefit/cost ratio [90, 91]. In other words, in future, for anti-cancer treatments to be considered excellent, evaluation of the cost (i.e., medical expenses) and toxicity (i.e., AEs) of each will be recommended, in addition to its benefit (antitumor efficacy); furthermore, it will be important to select overall treatments that are appropriate for the individual patient’s background and physical condition (Fig. 4). In this context, to judge the value of PD-1 pathway inhibitors, there is an urgent need to establish patient selection methods based on biomarkers, permitting prediction of the efficacy and AEs of PD-1 pathway inhibitors.

## Conclusion

More than 20 years after the discovery of PD-1 [1], several studies have identified the clinical efficacy of PD-1 blockade against a wide spectrum of solid and hematological malignancies, opening the door to a strategy for the treatment of cancer. In addition, on the basis of reports of clinical findings obtained using PD-1 inhibitors, longstanding theories regarding cancer immune surveillance [92] and cancer immunoediting, including the mechanism of cancer immune escape [93, 94], have recently been confirmed by fundamental research on tumor immunology. Nevertheless, a great deal of fundamental, exploratory research remains to be done on areas such as predictive biomarkers for therapeutic efficacy and adverse drug reactions. In future, we anticipate that clinical studies of PD-1 inhibitors and deep reverse-translational programs involving molecular and genomic research will reveal fundamental details of PD-1/PD-L1 signaling.
